# Challenges for Field-Effect-Transistor-Based Graphene Biosensors

**DOI:** 10.3390/ma17020333

**Published:** 2024-01-09

**Authors:** Takao Ono, Satoshi Okuda, Shota Ushiba, Yasushi Kanai, Kazuhiko Matsumoto

**Affiliations:** 1 SANKEN, Osaka University, 8-1 Mihogaoka, Ibaraki, Osaka 567-0047, Japan; 2 High Frequency & Optical Device Works, Mitsubishi Electric Corporation, 4-1 Mizuhara, Itami, Sendai 664-8641, Japan; 3 Murata Manufacturing Co., Ltd., 1-10-1 Higashikotari, Kyoto 617-8555, Japan; 4 International Center for Synchrotron Radiation Innovation Smart, Tohoku University, 2-1-1 Katahira, Aoba-ku, Sendai 980-8577, Japan

**Keywords:** graphene-FET biosensor, surface modification, Debye screening, nonspecific adsorption

## Abstract

Owing to its outstanding physical properties, graphene has attracted attention as a promising biosensor material. Field-effect-transistor (FET)-based biosensors are particularly promising because of their high sensitivity that is achieved through the high carrier mobility of graphene. However, graphene-FET biosensors have not yet reached widespread practical applications owing to several problems. In this review, the authors focus on graphene-FET biosensors and discuss their advantages, the challenges to their development, and the solutions to the challenges. The problem of Debye screening, in which the surface charges of the detection target are shielded and undetectable, can be solved by using small-molecule receptors and their deformations and by using enzyme reaction products. To address the complexity of sample components and the detection mechanisms of graphene-FET biosensors, the authors outline measures against nonspecific adsorption and the remaining problems related to the detection mechanism itself. The authors also introduce a solution with which the molecular species that can reach the sensor surfaces are limited. Finally, the authors present multifaceted approaches to the sensor surfaces that provide much information to corroborate the results of electrical measurements. The measures and solutions introduced bring us closer to the practical realization of stable biosensors utilizing the superior characteristics of graphene.

## 1. Introduction

The outstanding physical properties of graphene have led to a wide range of its applications in diverse fields such as high-speed and low-power-consumption operation in electronics [1,2,3,4,5,6,7,8], sensing over a wide range of wavelengths in photonics [9,10,11,12,13,14,15,16,17,18], and high-capacity rechargeable batteries for energy storage [19,20,21,22,23]. These applications include biosensing applications involving the measurement of chemical and biological substances [24,25,26,27]. High-sensitivity measurements of disease biomarkers and pathogens enable the early diagnosis of diseases and health monitoring [28,29]. In particular, diagnosis using samples such as saliva or sweat, which can be collected easily using minimally invasive methods, allows for continuous monitoring. However, the concentration of the target in such samples is generally low and requires high sensitivity for detection. Graphene has the potential for such sensitive detection and has been applied to a wide variety of detection targets over the past 15 years, starting with the earliest research studies [30,31]. Such targets range from ions [32,33,34], gases [35,36,37], organic molecules [38,39], nucleic acids [40,41], and proteins [42] to viruses [43,44,45], bacteria [30,46], and cells [47,48]. The detection mechanisms of graphene biosensors are also diverse, including those based on electrical methods, such as those using field-effect transistors (FETs) [49,50] and electrochemical techniques [51,52,53], and optical methods, such as the use of molecular beacons [54,55], chemiluminescence assays [56,57], surface plasmon resonance spectroscopy [58,59], and Raman scattering spectroscopy [58,60]. Graphene materials used in sensors include pristine graphene [31,61], graphene oxide [30,62], reduced graphene oxide [63,64], graphene quantum dots [65,66], graphene nanoribbons [67,68], and composite materials mixed with graphene [69,70]. The graphene materials of various forms have their advantages and disadvantages. First, graphene oxide is easily functionalized but has a lower mobility than pristine graphene. When graphene oxide is reduced, it exhibits properties more similar to those of pristine graphene. Graphene nanoribbons have the potential to increase sensitivity by opening the bandgap and improving the on/off ratio but are difficult to synthesize and process. Composite materials can be expected to acquire new properties, and their specific surface area can be further increased by three-dimensionalization, similarly to metal–organic frameworks [71]; however, this results in the loss of the inherent two-dimensional structure of graphene and reduced carrier mobility. Furthermore, the formats of sensors range from simple single-layer graphene on a chip to flexible and wearable sensor arrays that take advantage of graphene’s flexibility [72,73,74,75].

Graphene biosensors have been studied vigorously, and several venture companies have been established. However, graphene biosensors have yet to be widely used in society [76,77]. This situation is due to several problems, which are also being intensively investigated. This review focuses particularly on FET-based graphene biosensors (hereafter, graphene-FET biosensors), highlighting their advantages and the problems that hinder their practical applications. After providing an overview of the sensing mechanism of graphene-FET biosensors in Section 2, the authors discuss the first problem, Debye screening, in Section 3. The Debye screening problem reveals that the response of graphene-FET biosensors is strongly dependent on phenomena occurring at the nanoscale interface. Understanding and controlling the phenomena at the nanoscale interface is not easy. Another problem is the complexity of the sample components acting at such a nanoscale interface, i.e., the detection targets as well as nontargets such as ions and proteins. This complexity makes the understanding of the phenomena at the nanoscale interface even more difficult. This problem is most clearly observed in the nonspecific adsorption mentioned in Section 4, which often results in a decrease in signal-to-noise ratio to the point where the signal is no longer detectable. In Section 5, the authors list some technologies and findings of analyses of the phenomena occurring at the nanoscale interface of the sensor surface. However, such technologies and findings have remained insufficient thus far. As a result, we have not been able to successfully explain and control the sensor response. The authors believe that this hinders the stable operation of graphene-FET biosensors, which is a prerequisite for their social implementation. In the following, these problems and some of their solutions will be discussed.

## 2. Overview of Graphene Biosensing by Field Effects

**Figure 1:**
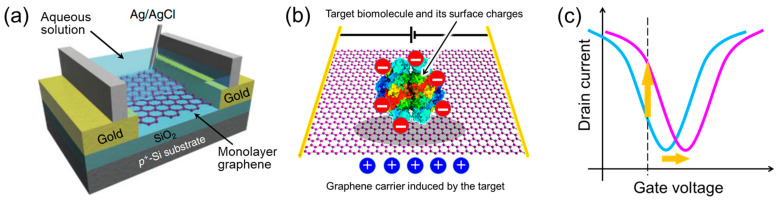
Schematics of graphene-FET biosensors. (**a**) Schematic of a typical graphene-FET biosensor. The sample solution is placed in a silicone rubber barrier (light gray). Cited from [81]. Copyright 2011 The Japan Society of Applied Physics. (**b**) Electrostatic interaction between the graphene channel and target molecule. (**c**) Transfer characteristics of the graphene-FET biosensor. Following the hole induction in the graphene, the drain current–gate voltage curve shifts horizontally in the positive direction (from blue line to pink line), and the hole current increases at a fixed gate voltage (dotted line).

**Figure 2:**
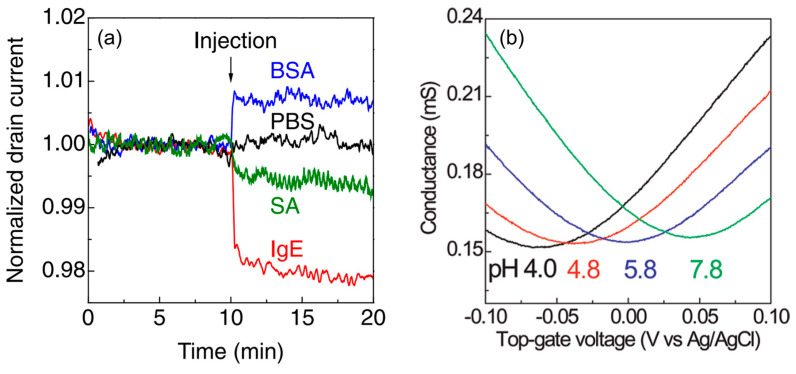
Electrical responses of graphene-FET biosensors without surface modification due to protein surface charge and pH. (**a**) Drain–current time courses of graphene-FET biosensors with various adsorbed proteins. The hole current was decreased by adsorption of immunoglobulin E (IgE) and streptavidin (SA) proteins and increased by adsorption of BSA protein. Phosphate-buffered saline (PBS) was the control; thus, no protein was injected. (**b**) Gate voltage–conductance characteristics in solution pHs of 4.0 to 7.8. Cited from [31,81]. Copyright 2011 The Japan Society of Applied Physics and 2009 American Chemical Society.

Figure 1a shows a schematic of a typical graphene-FET biosensor. The source and drain gold electrodes are connected to a graphene channel immersed in a sample solution. Through these electrodes, a drain voltage is applied to the graphene channel, driving the carriers in graphene. A gate voltage is applied to the graphene channel through the aqueous solution from the gate electrode to modulate the carrier density of graphene. A Ag/AgCl reference or Au or Pt pseudo-reference electrode is often used. When a detection target with surface charges is adsorbed on graphene, carriers with the opposite sign to the surface charges are induced in graphene, resulting in changes in the transfer characteristics of the graphene-FET biosensor (Figure 1b). The transfer characteristics of graphene FETs are different from those of silicon FETs. Carriers in graphene switch between holes and electrons across the minimum current (charge neutrality point). For example, when a negatively charged material is adsorbed on graphene and hole carriers are induced in graphene, the hole current increases and the transfer characteristics shift in the positive direction of the gate voltage (Figure 1c) (other models have also been proposed as described later). The change in hole current when several types of protein are adsorbed is shown in Figure 2a. Each protein has a different isoelectric point. For example, the isoelectric point of bovine serum albumin (BSA) is 5.3, and BSA is negatively charged at pH 6.8. Therefore, adsorbed BSA induces holes in graphene at that pH. This explanation is consistent with the results in Figure 2a, where the hole current increase is measured. The reasons for the superiority of graphene-FET biosensors based on this mechanism can be summarized as follows:

1. Graphene has a high specific surface area because all its carbon atoms are present on the surface [78,79]. In addition, as it is a carbon material, it has a wide potential window [80]. Therefore, unlike silicon FET biosensors, which require a SiO_2_ insulating layer, the target in the aqueous solution is in direct contact with the graphene channel, inducing carriers effectively.

2. Very few carriers are induced by small amounts of target molecules. However, owing to the high carrier mobility of graphene [82,83], a small modulation of carriers can be converted to a large current change. In addition, the current-based detection method allows for label-free real-time sensing, unlike conventional optical detection methods.
3. Because the temporal changes in biosensor characteristics, such as current and the charge neutrality point, are monitored, an on/off ratio of the FET is not needed for the biosensing. The lack of a bandgap, which is the greatest weakness of graphene in semiconductor applications, is not an issue in graphene-FET biosensors [6,84,85].

Indeed, graphene is one of the best electrical biosensor materials available.

pH has been measured using graphene-FET biosensors (Figure 2b) [25,31,86]. The reported pH sensitivities, however, widely range from the lack of sensitivity to the sensitivity exceeding the Nernst limit [87,88,89]. Moreover, several mechanisms of pH sensing have been proposed [90,91,92,93,94]. For example, for the most well-known site-binding model, it is considered that the few oxygen-containing defects in graphene serve as binding sites for protons. Changes in electric double-layer capacitance, changes in solution potential, and other effects can be attributed to the response to pH. Importantly, these mechanisms are not mutually exclusive but are considered to act in combination. Therefore, the problem is not so much which mechanism is correct, but rather the difficulty of constructing a model, that is, a combination of mechanisms that comprehensively explains the wide variation of experimental results. This problem is due to the fact that the structure of graphene-FET biosensors has not been sufficiently clarified. The structure here is a set of elements such as the covalent bonds in graphene (whether it has oxygen-containing groups or not, and if it has groups, how dense the groups are), the impact of the supporting substrate on graphene, the contribution of non-proton ions in the solution, and others. It is currently difficult to control and understand all of these elements, and this has led to the controversy regarding pH sensing. The site-binding model was originally proposed as a model for silicon FET-based pH sensors, one of the few examples of FET-based biosensors in widespread use [95]. When the structure of the graphene-FET biosensors is well clarified in the near future, the authors expect that the site-binding model will largely explain the characteristics of pH sensing using graphene-FET biosensors.

To provide graphene-FET biosensors with higher selectivity and binding specificity, it is necessary to modify graphene with various molecules (receptors) that bind specifically to the detection target. There are many reports on the surface functionalization of graphene with ionophores [33,96], other organic molecules [97,98,99], biomolecules [100,101,102], and nanoparticles [103,104,105]. In particular, graphene oxide can be easily functionalized with various molecules through covalent bonding with its oxygen-containing groups [106,107,108]. On the other hand, because the surface of pristine graphene is inert, linker molecules are widely used to achieve such modifications. The most commonly used linker molecule is 1-pyrenebutanoic acid succinimidyl ester (PBASE) [81,109,110]. This molecule has a pyrenyl group that binds to graphene through π–π interactions without breaking the intrinsic honeycomb structure of graphene, which is the key to graphene properties such as carrier mobility. Moreover, it has a succinimide group that covalently bonds to the amino groups of many receptor molecules, enabling the modification of the receptor molecule to graphene. In addition, because the receptor molecules bind to these linkers at only one or a few points, functions of the receptors such as binding affinity to the detection targets are expected to be preserved compared with when they are adsorbed directly onto graphene. Linker molecules with similar structures have also been reported [111,112].

**Figure 3:**
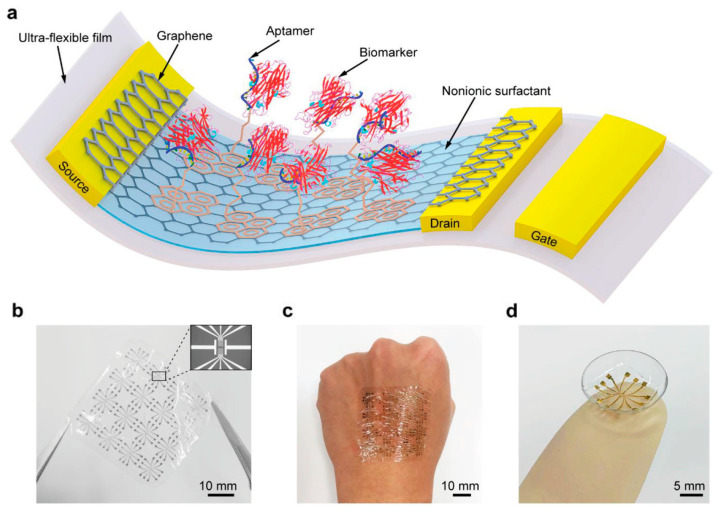
Flexible graphene-FET biosensor modified with aptamer via PBASE linkers. (**a**) Schematic of the biosensor. The aptamer captures tumor necrosis factor-α, an inflammatory cytokine biomarker. (**b**–**d**) Photographs of the free-standing biosensor (**b**), the biosensor mounted on the human hand (**c**), and the contact lens (**d**). Cited from [124]. Copyright 2019 John Wiley & Sons, Inc.

There have been reports of specific detection by modifying receptor molecules such as aptamers and glycans via linkers (Figure 3). Aptamers are single-stranded DNA, RNA, or other nucleic acids that have binding affinity to specific biomolecules. There is also a class of aptamers called peptide aptamers that use amino acids instead of nucleic acids [113,114,115,116]. Compared with antibodies which are widely used, aptamers are smaller and chemically more stable. Glycans, on the other hand, have fewer applications to sensors than aptamers, and their binding affinity is generally lower than those of antibodies and aptamers; however, they are as small as aptamers and function as receptors in many biological events [117,118]. For example, when influenza viruses infect cells, the viruses bind to sialoglycans on the cell surface and initiate infection [119,120,121]. During this process, the viruses recognize slight differences between human and avian sialoglycan ends. Avian influenza viruses bind only to the sialoglycan ends of avian origin, whereas human infectious influenza viruses bind to the sialoglycan ends of human origin. However, there was no sensitive method to examine the virus binding affinity to sialoglycans [122]. Viruses can be detected on the basis of their infectivity by modifying sialoglycan receptors on graphene-FET biosensors. In a proof-of-concept study using a lectin protein that exhibits similar binding affinities to influenza viruses [123], the pseudo-human influenza virus (lectin derived from *Sambucus sieboldiana*) and pseudo-avian influenza virus (lectin derived from *Maackia amurensis*) were detected within 10 min at concentrations as low as 130 pM and 150 pM, respectively. Cross-reactivity was almost nonexistent.

## 3. Debye Screening

Debye screening is one of the most severe problems with graphene-FET biosensors and other FET-based biosensors [125,126,127,128,129]. Debye screening is a phenomenon in which ions in an aqueous solution aggregate around the detection target and neutralize its charge, making the target undetectable. The Debye screening length *λ*_D_, which is the characteristic screening length, is expressed as

(1) $$\lambda_{D}=\sqrt{\frac{\varepsilon RT}{2F^{2}I}},$$

where *ε*, *R*, *T*, *F*, and *I* are the permittivity, gas constant, temperature, Faraday constant, and ionic strength, respectively. The Debye screening length under physiological conditions is approximately 1 nm or less [130,131]. On the other hand, the total length of an immunoglobin G (IgG) molecule, which is an antibody commonly used as a receptor, is about 15 nm [132]. This strongly suggests that the target captured by IgG is away from graphene beyond the Debye screening length and is, therefore, undetectable. To extend the Debye screening length, it is common practice reducing the ionic strength [133,134,135,136,137,138], but this is undesirable because it impairs the buffering capability of the aqueous solution and the reactivity of the biomolecules, and destabilizes the electric field around graphene.

**Figure 4:**
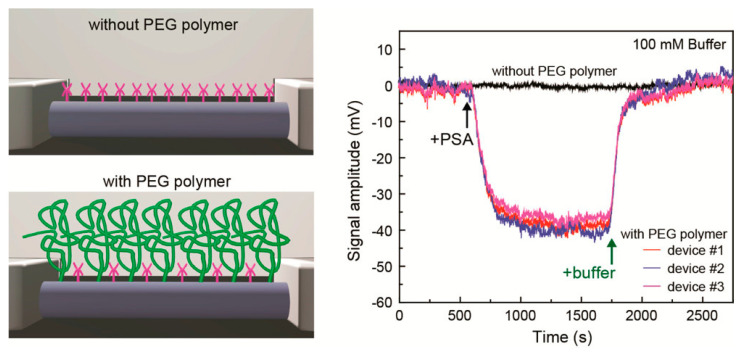
Extension of the Debye screening length and increasing the sensitivity by PEG modification on the biosensor. A prostate-specific antigen (PSA) biomarker in 100 mM buffer was detected. This figure shows an example of silicon nanowire biosensors; see [155] for an example of graphene-FET biosensors. Cited from [149]. Copyright 2015 American Chemical Society.

Numerous efforts have been made to address the problem of Debye screening. For example, receptor molecules smaller than IgG have been used, such as aptamers [139,140,141], fragment antibodies [142,143,144], and nanobodies [145,146,147]. Fragment antibodies are fragments of the binding end of an antibody such as IgG, whereas nanobodies are antibodies derived from animals such as camels and are only about 1/10th the size of normal IgG. A unique evolution of the aptamer-based method is to induce the conformational changes of aptamers to which the target binds and to bring the charge of the aptamer in close proximity to the sensor surface [131]. The detection of fM-order dopamine and serotonin in PBS and artificial cerebrospinal fluid has been reported. This method can be applied to electrically neutral detection targets by detecting the charge of the deformed aptamer [148]. This method makes good use of the aptamer, which can be designed in a bottom-up manner. The appropriate modification of the sensor surface to detect targets farther than the Debye screening length has also been reported. Sensors modified with poly(ethylene glycol) (PEG) along with receptors have been reported to detect targets on the order of nM in 100 mM phosphate buffer or PBS, which is comparable with physiological salt concentrations (Figure 4) [149]. The change in the dielectric constant near the sensor surface may have contributed to this sensing mechanism. Finally, a technique to mitigate the effects of Debye screening by applying a high-frequency electric field in the kHz to GHz range has also been reported [150,151,152]. By applying a 2 GHz radiofrequency field through graphene, one can detect 1 nM streptavidin protein trapped beyond the Debye screening length in PBS [152]. This solution to the Debye screening problem takes advantage of the high carrier mobility of graphene [153,154].

**Figure 5:**
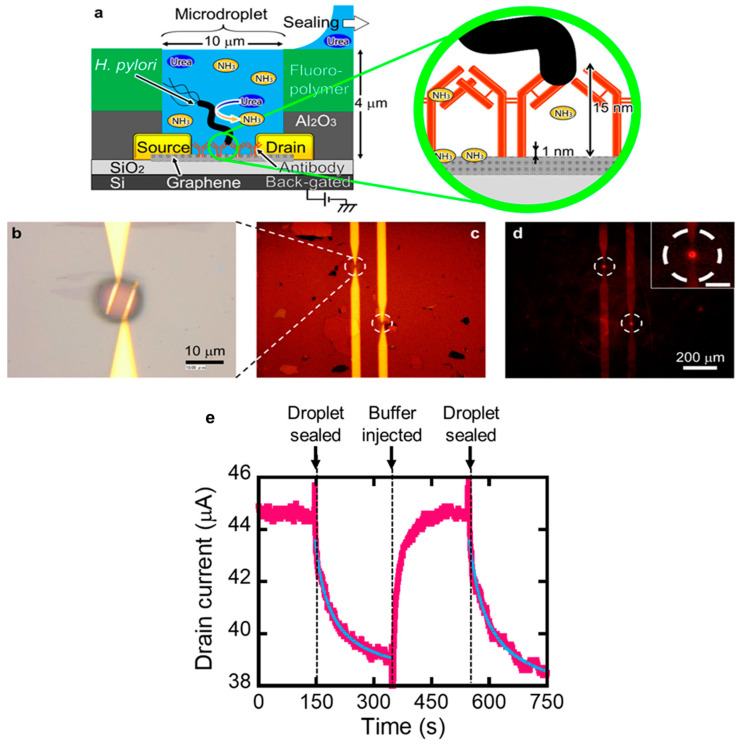
Graphene-FET biosensor with a microwell. (**a**) Schematic of the detection mechanism. The target, *Helicobacter pylori*, is captured by the IgG antibody, and the ammonia produced by *H. pylori* is concentrated in a microdroplet for detection. (**b**,**c**) Bright field images of the device. (**d**) Fluorescent image of the device encapsulating rhodamine 6G dye. The inset shows droplet formation. (**e**) Response of a graphene-FET biosensor through the reaction of urease immobilized on graphene in a microdroplet. Cited from [159]. Copyright 2019 American Chemical Society.

In addition to these methods, the problem of Debye screening can be solved by detecting the reaction products generated by the detection target (Figure 5) [156,157,158,159]. Even if the target is away from graphene beyond the Debye screening length, it can be detected if its reaction products are adsorbed directly on graphene. Many gas species are adsorbed directly onto graphene and detected electrically [35,160,161,162,163,164,165,166]. If the target produces the gas molecules, it can be detected by gas sensing. However, such small gas molecules will diffuse freely into aqueous solutions. Therefore, it is necessary to keep the gas molecules around graphene. If the reaction product, the gas, is confined in a microwell fabricated on graphene, the gas can be concentrated on the graphene. Such a technique is used in digital biosensing, a highly sensitive method for the detection of single-molecule enzymes [167,168,169,170,171,172]. In digital biosensing, even a minute amount of a reaction product from a single enzyme molecule reaches a high concentration that can be detected by confining and accumulating the product in a microwell.

A microwell was formed on graphene using semiconductor microfabrication technology [173,174,175,176]. Briefly, an Al_2_O_3_ layer and a fluoropolymer layer were formed in sequence on graphene, and the fluoropolymer was dry-etched using oxygen plasma after photolithography. The Al_2_O_3_ layer protected the graphene from the plasma. The exposed portions of the Al_2_O_3_ layer were then removed by wet etching, exposing the graphene in the microwell (Figure 5a). When a microdroplet was formed in the microwell, the ammonia-producing enzyme urease, which was the detection target fixed on graphene, was encapsulated in the microdroplet. Ammonia, which acts as an electron donor [160], was produced and concentrated on the graphene, resulting in a decrease in the hole current of the graphene-FET biosensor. When the microdroplet broke up and the ammonia contents diffused to the outside of the microwell, the current returned to its original value. Note that ammonia continued to be produced even at this time as urease was fixed on graphene.

Because the very small amount of urease is anchored on graphene, ammonia reaches a detectable concentration only when the reaction volume is highly limited by the microdroplet.

*H. pylori*, the pathogenic bacterium responsible for gastric cancer, survives in the stomach because it has urease and can neutralize stomach acids with ammonia [177,178,179]. *H. pylori* and its urease were trapped on graphene with IgG and encapsulated in a microwell with a volume of 314 fL. Ammonia production by urease at a reaction rate of 540 zmol/s (100 μM/min) was successfully detected. This reaction rate is equivalent to 0.04 *H. pylori* cells, which is much less than one bacterial cell, indicating that detection was possible at the level of bacterial fragments. This new method detected *H. pylori* in a 230 mL^−1^ sample within 30 min. This is 10^5^ times more sensitive and can detect faster than commercially available test kits.

## 4. Attempt to Rationally Extract Signals from Samples with Complex Components

**Figure 6:**
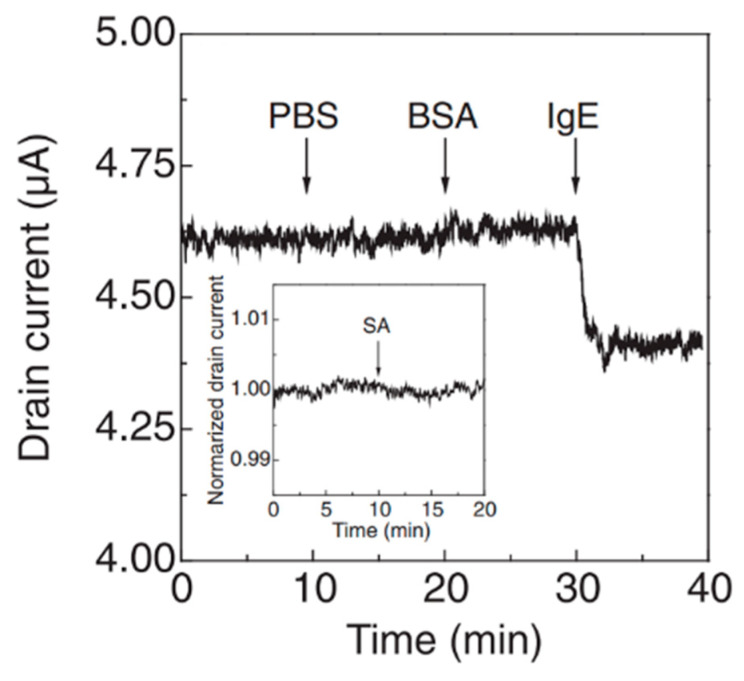
Selective detection of IgE protein using graphene-FET biosensor modified with an IgE-specific aptamer. The nonspecific adsorption of BSA and SA proteins was suppressed because the graphene surface was blocked with BSA before the measurement (PBS is the control). Cited from [81]. Copyright 2011 The Japan Society of Applied Physics.

Graphene-FET biosensors are extremely sensitive to the surrounding environment, which leads to high detection sensitivity; however, they are also sensitive to nontargets such as ions and proteins. This makes it particularly difficult to interpret the response of graphene-FET biosensors to clinical samples. A number of countermeasures have been proposed to address this major practical problem. The blocking of the graphene surface is the most commonly used measure, and many blocking reagents have been applied, including commercial products [180,181,182,183,184,185]. For example, proteins such as BSA [186,187], surfactants [188,189], and PEGs [190,191] are often used (Figure 6). These reagents occupy binding sites on the sensor surface where molecules can bind nonspecifically, and they also hydrophilize the surface to inhibit nonspecific adsorption due to hydrophobic interactions. As the surface of graphene is hydrophobic [192,193], nonspecific adsorption due to hydrophobic interactions must be considered more than in other conventional sensors with hydrophilic (e.g., SiO_2_) surfaces. The purification of samples is also effective [103,194]. The purification often requires large equipment such as centrifuges, but there have been vigorous attempts to integrate a purification unit onto the biosensor chip [195,196,197]. In contrast to passive methods to prevent nonspecific adsorption from occurring as described above, methods to actively remove nonspecifically adsorbed molecules have also been proposed. Such methods mainly involve the application of shear stress to the biosensor surface using an alternating electric field, mechanical vibration, or microfluidic devices [198,199,200]. Another problem is contamination by residues of poly(methylmethacrylate), photoresist, and other materials used in the fabrication of graphene-FET biosensors [201,202,203,204,205]. Although many cleaning methods have been reported, such as annealing under a hydrogen atmosphere, there is still no method to completely clean contaminated surfaces. Surface contamination by such polymer residues prevents surface modification by small molecule linkers in particular.

In addition to the problem of such foreign substances and surface contaminations, another problem is that a target biomolecule has complex surface structures [206]. The surfaces of biomolecules have a mixture of positively and negatively charged areas. Because these areas are often larger than the Debye screening length, the carriers induced by the same biomolecule will be different depending on the orientation of the biomolecule contacting with graphene. Furthermore, several models have been proposed with regard to the mechanism by which the target molecule alters the transfer characteristics of graphene-FET biosensors. The most frequently mentioned model is the above-mentioned model of electrostatic interactions, in which the surface charge of the molecule induces carriers with opposite signs (Figure 1b,c) [207]. Other models include a model of direct carrier transfer from the target molecule to graphene [208], which the detection of electrically neutral gas molecules is based on, and a model in which the molecule is the scattering source of the carriers of graphene [209]. These mechanisms can simultaneously induce opposite responses to graphene, making the interpretation of graphene-FET biosensor responses difficult. The authors believe that this difficulty has led to arbitrary interpretations and the lack of reproducibility of graphene-FET biosensor results, which are obstacles to the practical applications of graphene-FET biosensors.

**Figure 7:**
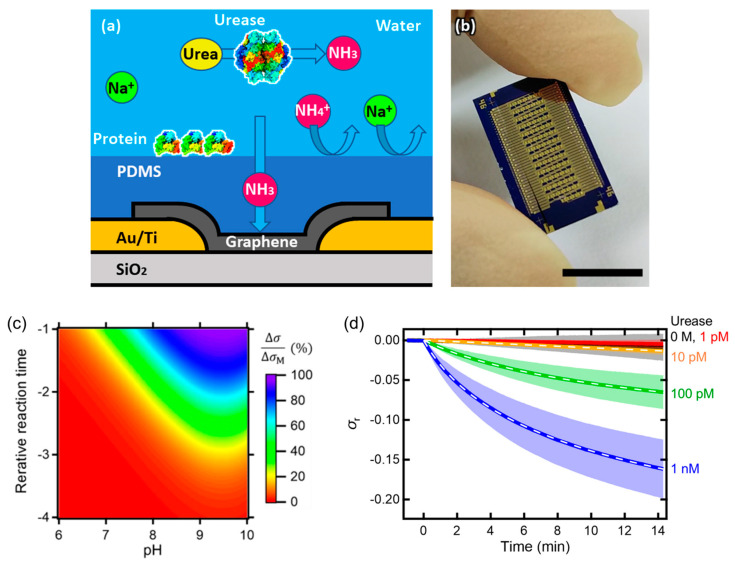
PDMS-coated graphene-FET biosensor. (**a**) Schematic and (**b**) photograph of the biosensor. A total of 82 graphene-FETs were integrated on a chip. (**c**) Simulated response of the biosensor for urease reaction. The response can be measured in the shortest reaction time at pH 9.5. (**d**) Measurement results of urease reaction at pH 9.5. Almost perfect agreement with the fit obtained using the model equation (white dotted line). Cited from [212]. Copyright 2023 The Japan Society of Applied Physics.

The aforementioned problems can be solved by restricting the molecular species that can reach the graphene surface to only gas molecules. This restriction can be achieved by coating graphene with a gas-permeable poly(dimethylsiloxane) (PDMS) membrane (Figure 7). The ions and proteins present in the solution are blocked by the membrane and do not reach the graphene surface, thus not affecting the sensor response. This surface blocking is different from blocking with ordinary blocking reagents because PDMS is hydrophobic and does not allow aqueous solutions to penetrate, and even the adsorption of ions on graphene is eliminated. The isolation of graphene from the aqueous solution by the PDMS membrane also reduces electrical noise. On the other hand, gas molecules, as the detection target, penetrate through the membrane and reach the graphene surface [210,211]. Thus, the signal-to-noise ratio could be improved by a factor of around 1000 compared with that without the membrane. Because the electrical response of the PDMS-coated graphene-FET biosensor is considered solely due to the detection target because of its high signal-to-noise ratio, the response can be described by a simple mathematical model. For urease detection in solution, the model can be broken down into the following three factors: 1. the enzymatic production of ammonia in solution based on the Michaelis–Menten kinetics; 2. the pH-dependent dissociation equilibrium of ammonia with ammonium ions, which are intercepted by the membrane; and 3. the kinetics of the association and dissociation of the ammonia gas molecule (electron donor) and graphene surface. On the basis of these three factors, the response model of the graphene-FET biosensor to the urease reaction is described by the following two equations:

(2) $$\frac{d\sigma_{r}}{dt}=k_{on}\left[{NH}_{3}\right]\left(\frac{∆\sigma_{M}}{\sigma_{0}}-\sigma_{r}\right)-k_{off}\sigma_{r},$$

(3) $$-\frac{d[{\left({NH}_{2}\right)}_{2}CO]}{dt}=\frac{v_{max}[{\left({NH}_{2}\right)}_{2}CO]}{K_{M}+[{\left({NH}_{2}\right)}_{2}CO]},$$

where *σ*_r_ is the rate of change in conductivity relative to the initial conductivity *σ*_0_, *t* is the time, *k*_on_ is the binding rate constant, *k*_off_ is the dissociation rate constant, Δ*σ*_M_ is the maximum change in conductivity, *v*_max_ is the maximum reaction rate of urease, and *K*_M_ is the Michaelis constant of urease. Owing to the pH-dependent equilibrium, a measurement window exists in which ammonia is sensitively measured in the high-pH range. Similar to this window for ammonia measurement, there is another window for the pH dependence for enzyme reactions, and the two windows can be superimposed to derive the optimal condition for enzyme reaction measurements (Figure 7c). When the urease reaction was measured under this condition, the model explained the actual response well over a wide range of enzyme concentrations (Figure 7d). This leads to the rational model-based design of graphene-FET biosensors and reproducible responses of the biosensors. These are essential for the practical applications of graphene-FET biosensors.

## 5. Multifaceted Evaluation of Measurement Systems by Combining Multiple Measurement Principles

Graphene-FET biosensors basically measure the change in the carrier density of graphene attributable to the detection target as one of the changes in transfer characteristics. This scheme takes advantage of the high carrier mobility of graphene and has become a mainstream measurement method. On the other hand, by measuring other parameters, much information can be extracted from the detection target. This can also corroborate the results of mainstream measurement methods.

**Figure 8:**
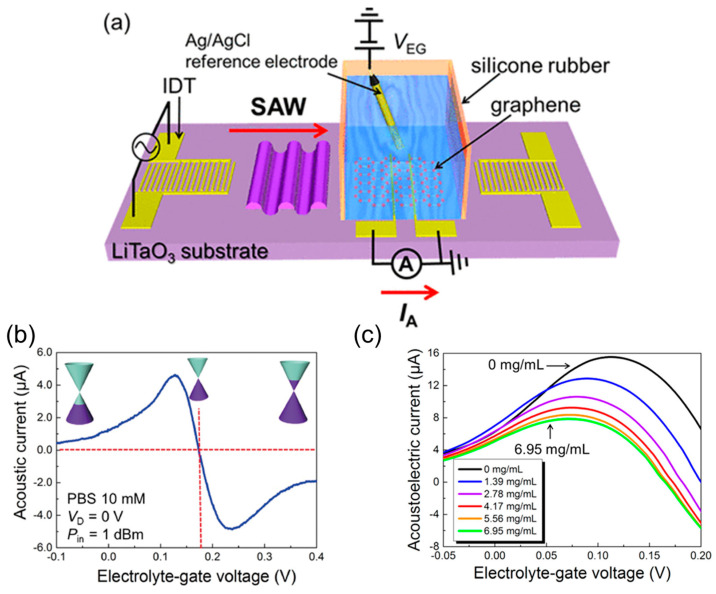
GSAW sensor. (**a**) Overview of the GSAW sensor. (**b**) Transfer characteristics of the GSAW sensor. (**c**) Enlarged view near the hole-side peak of the transfer characteristics when positively charged microbeads are introduced. Cited from [222,224]. Copyright 2016 The Japan Society of Applied Physics and 2017 American Chemical Society.

Carriers in graphene can be driven without the need to connect the metal electrodes directly to graphene. Such wireless graphene sensors are useful for wearable devices, continuous monitoring, and energy-saving sensor operation [213,214,215,216,217,218,219,220]. For example, wireless carrier transfer through a surface acoustic wave (SAW) has been demonstrated (Figure 8) [221,222,223]. The density of acoustic current flowing through a graphene channel fabricated on a piezoelectric substrate, *J*_AE_, is expressed as

(4) $$J_{AE}=-\frac{\mu I\Gamma }{v},\;\;\;\;\;\;\;\;$$

where *μ* is the carrier mobility of graphene, *I* is the wave intensity of SAW on graphene, *v* is the velocity of SAW, and Γ is the attenuation per unit length. The transfer characteristics of the graphene-FET biosensor driven by SAWs (GSAW sensor) are different from normal ambipolar characteristics (Figure 8b). This is because the positive and negative carriers are driven in the same direction by SAWs. The current switches between positive and negative values across the charge neutrality point, where the current is zero. Because the drain voltage is not superimposed, the gate voltage at the charge neutrality point is also different from the normal ambipolar characteristics. A GSAW sensor can detect the mass of the detection target as well as its surface charge. This is because the amplitude of the SAW is reduced by mass loading. When amino group-modified microbeads were introduced onto the GSAW sensor, both the positive charge of the amino groups and the mass of the beads acted on graphene. The hole density of graphene decreased in response to the positive charge, and the hole current peak shifted towards the negative gate voltage (Figure 8c). Simultaneously, the amplitude of the SAW decreased in response to the mass, and the hole current at the peak decreased. The SAW on biosensors can also be used to remove molecules that are nonspecifically adsorbed on the biosensor surface.

**Figure 9:**
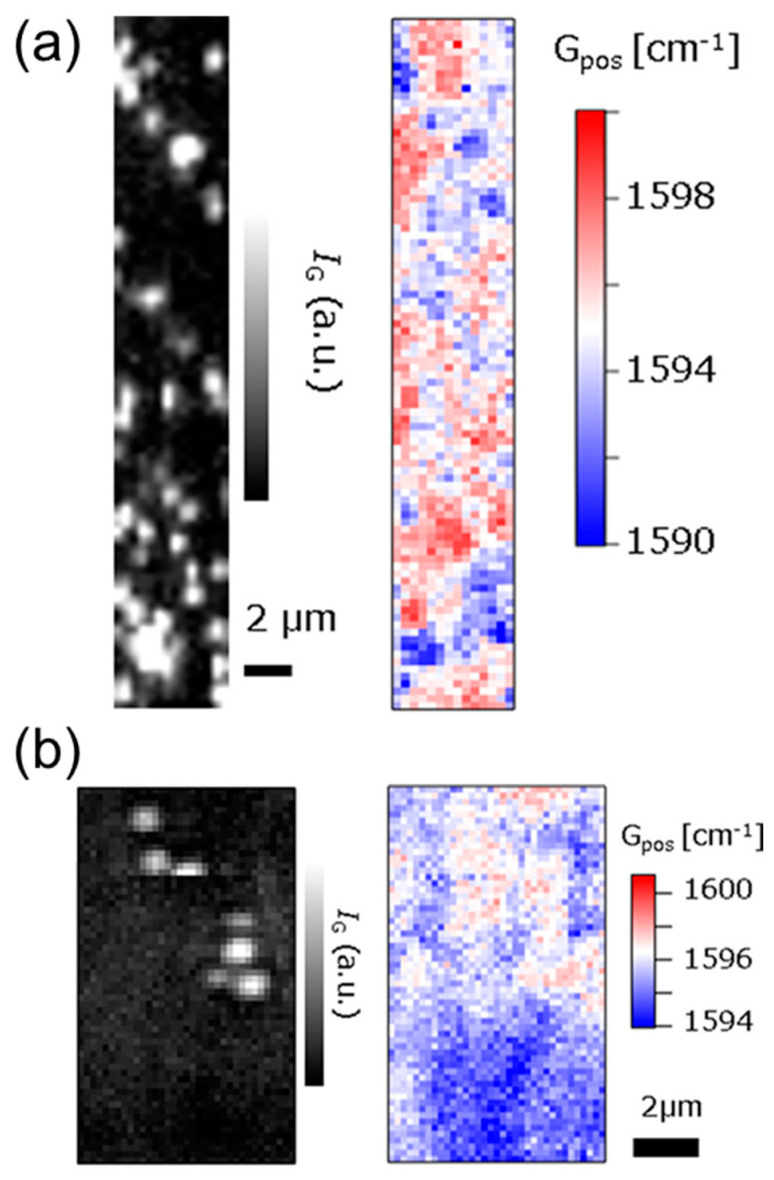
Raman mapping of graphene with (**a**) negatively and (**b**) positively charged microbeads. Gray and color images show the Raman intensity and peak position, respectively. Cited from [227]. Copyright 2018 American Chemical Society.

In GSAW sensors, the charge of detection targets is measured in terms of current, but it is also possible to measure the charge in terms of Raman scattering. Graphene exhibits strong Raman scattering, which varies depending on the surrounding environment [60,225,226,227,228,229]. The carrier density of graphene can be estimated spectroscopically as the Raman scattering peak shift. The frequency shift of the G peak of the Raman scattering of graphene is related to the electron density *n*_e_ in the following equation:

(5) $$n_{e}={\left(\frac{21\Delta G_{pos}+75}{\hbar \left|v_{F}\right|\sqrt{\pi }}\right)}^{2},$$

where Δ*G*_pos_ is the frequency shift of graphene from *G*_pos0_ = 1581 cm^–1^ and *ν*_F_ is the Fermi velocity. Here, polystyrene beads modified with negatively charged carboxyl groups or positively charged trialkylammonium groups were adsorbed on graphene, and Raman scattering peak shifts were mapped (Figure 9). The negatively charged beads, which reduce the electron density of graphene, were observed as a downshift of the G peak of the Raman scattering, whereas the positively charged beads were observed as an upshift of the same peak. Although current measurements can only determine the carrier density across the entire graphene channel, this mapping technique enables the visualization of the distribution and inhomogeneity of carriers in graphene.

These techniques corroborate the results of charge detection using electric current and will be one of the evaluation techniques for the development of graphene-FET biosensors. In addition to Raman scattering, many other sensing techniques based on the optical properties of graphene have been reported [230,231,232,233,234,235], and the combined use of these techniques with current measurements improves the reliability of the measurement techniques.

## 6. Conclusions and Outlook

In this review, the authors introduced the mechanisms underlying the advantages of graphene-FET biosensors and discussed some of the challenges that hinder their practical applications and possible solutions to these challenges. The high specific surface area and high carrier mobility of graphene make this material the best biosensor candidate. The modification of the receptor molecule is necessary to obtain a biosensor with a high degree of selectivity. Improving the binding affinity of the receptor to the detection target and the durability of the receptor for continuous measurement will be a future issue. The process and system design for the continuous operation of graphene-FET biosensors in clinical samples will also be necessary in the future.

Regarding the Debye screening problem, numerous measures have been proposed to modulate the electrical properties of the solid–liquid interface. They will require future technological maturation to stably induce desirable changes at the nanoscale interface. For other measures that use the microwell, it is necessary to construct measurement systems that enable automated fluid handling.

Surface blocking and sample purification are important in the prevention of nonspecific adsorption. Even with these techniques, it is difficult to completely eliminate nonspecific adsorption; thus, effective washing and cleaning techniques are also necessary at the next stage of biosensor development. These techniques are even more important for clinical samples containing large amounts of nontarget substances. The mechanical strength of graphene against shear stress will become a problem [236]. A method that detects the reaction product of the target rather than the target itself, or the enzyme immunoassay based on this methodology, has the potential to solve the problem [237].

A deeper understanding of the sensing mechanism and improvement of the reliability of the obtained results can be achieved through a multifaceted evaluation of sensing systems by combining multiple measurement principles. However, it is not easy to put the biosensor systems using multiple methods into practical or commercial use because it complicates the configuration of the measurement device. Rather, it is useful to corroborate measurement results in the research and development of graphene-FET biosensors. For the improvement of the reliability and accuracy of these biosensors in practical or commercial use, a realistic option is to form an array of graphene-FETs on a biosensor chip and to average a large number of measured data. Finally, the practical applications of graphene-FET biosensors will require low-cost, mass-producible device formats and a compact, user-friendly measurement apparatus.

## Data Availability

No new data were created or analyzed in this study.
